# New Hampshire Veterinary Medical Association

**Published:** 1896-09

**Authors:** L. Pope

**Affiliations:** Secretary


### NEW HAMPSHIRE VETERINARY MEDICAL ASSOCIATION.

The eleventh meeting was held at Concord, July 14, 1896, with Dr. Lilico in the chair. Drs. Tuttle, Wilkinson, Lilico, Abbott, Macguire, Hart, and Pope responded to roll-call.

Dr. Wiggins's application was presented and laid over until the next meeting.

Dr. Lilico then addressed the Association regarding the subject of veterinary legislation.

After a long discussion it was voted that a committee be appointed to draw up a bill and present it at the next meeting. Said bill to be similar to those of other States having examining boards.

After a discussion on tuberculosis, the meeting adjourned until September 1st.

L. POPE, JR., M.D.V.,
Secretary.

